# A Comparative Analysis of Public Hospital Pharmacy Systems in Norway and Pakistan: A Pilot Implementation of the American Society of Health-System Pharmacists’ (ASHP) Guidelines

**DOI:** 10.3390/ijerph19137885

**Published:** 2022-06-27

**Authors:** Bilal Hasan Hashmi, Adnan Kisa

**Affiliations:** 1 Institute of Health and Society, University of Oslo, 0373 Oslo, Norway; bilalhashmi047@gmail.com; 2 School of Health Sciences, Kristiania University College, 0152 Oslo, Norway; 3 Department of International Health and Sustainable Development, School of Public Health and Tropical Medicine, Tulane University, New Orleans, LA 70118, USA

**Keywords:** Pakistan hospital pharmacy practice, Norwegian hospital pharmacy practice, hospital pharmacists

## Abstract

The objective of the study was to analyze and compare public hospital pharmacy practices in Pakistan and Norway. In addition, the study intended to identify the challenges to improving the public hospital pharmacy system and to propose recommendations that could help reform the practice to enhance patient safety and compliance. A cross-sectional study was conducted to understand public hospital pharmacies’ organizational structure and determine their practices in Norway and Pakistan. The results of the research showed differences in 11 main areas of the pharmacy systems of the sampled hospitals. When compared to Norway, the study found that the public hospital pharmacy system in Pakistan could be improved in nine main areas. The results show that hospital pharmacies in Pakistan could benefit from the experience of similar international structures in relation to universal standards and practices.

## 1. Introduction

For decades, pharmacists have made significant contributions to elevate their profession in hospitals worldwide [1,2,3,4,5]. There has been a long tradition of clinical activities being performed by pharmacists within the hospital setting. A large body of research has discovered a positive impact on patient outcomes [6,7]. For instance, an assessment of pharmacists’ clinical involvement from 1989 to 1998 showed a reduced number of deaths with the delivery of different pharmacy services in seven main areas. These areas were drug use evaluation, in-service education, monitoring of ADRs, management of drug protocols, participation in the cardiopulmonary resuscitation team, participation in medical rounds, and completion of admission drug histories [6].

A study on the role of hospital pharmacists showed that their presence in hospital settings had improved the effectiveness of drug therapy in different patients [6]. To capitalize on these successes, hospital pharmacy organizations worldwide are calling on their members to expand their focus from the distribution of medications to patient outcomes [8]. For example, the development of the Basel Statement and the Six Goals promoted in Vision 2015 by the International Pharmaceutical Federation and the Canadian Society of Hospital Pharmacists accentuate the importance of patient outcomes [9]. This new emphasis is consistent with the vision statement by the pharmacy profession in Canada: “Optimal drug therapy outcomes for Canadians through patient-centered care” [10].

In 2009, hospital pharmacy professionals gathered in Basel, Switzerland, to expand on the vision statement of the hospital pharmacy system. The latest changes to patient-centered practice now involve pharmacists giving recommendations to the prescribing physicians as well as offering consultations to patients or their caregivers. As pharmacists are incorporated into the healthcare team, their responsibilities for preparing and dispensing medications have been expanded to improving pharmacotherapeutic strategies of patients and being answerable for healthcare consequences [4]. In evidence-based practice, the hospital pharmacist seeks to manage as well as shape the standard and efficacy of every phase of the procedures used in medicating. Professional pharmacy practice follows several important outlines of training as well as education, inter-professional associations, clinical practice, and administrative authority [11]. A substantial number of researches have investigated hospital pharmacy practice, the competence of pharmacy services, and their staff [12,13,14,15]. These studies concentrate on practices in a particular area or a country or on particular pharmacists or hospitals.

Practices among pharmacies within hospitals differ from one country to another, even though many regions have the same issues [16]. In many nations, the responsibilities of a hospital pharmacist have evolved, with the latest change being from a drug-oriented service to a patient-oriented one. Both the American Society of Health-System Pharmacists (ASHP) and the European Association of Hospital Pharmacists have published surveys on ongoing practices and guidelines in institutional pharmacy services [5,17]. However, these studies have been limited to hospitals in the United States. Some researchers have compared the organizational structure of hospital pharmacies in France and Canada [15]. These studies have helped improve the overall quality of healthcare systems.

The Norwegian Ministry of Health and Care Services and the Pakistan Ministry of National Health Services Regulations and Coordination are the leading legislative authority, including pharmaceuticals and hospital pharmacy practice. However, many errors have recently occurred in hospital pharmacies in Pakistan [18,19,20,21,22]. This raises the question of how the organizational structure of hospital pharmacies in Pakistan differs from developed countries, such as Norway, and how these differences can produce considerable variations in the outcomes of pharmaceutical care. When the literature is reviewed, there appears to be a need to examine hospital pharmacy systems of developing countries and compare these with the best practices. Accordingly, this pilot study surveys the organizational structure of public hospital pharmacy systems in Norway and Pakistan and compares their practices. The research question is: “What are the differences in the organizational structure and practices of public hospital pharmacies in Norway and Pakistan?”

In order to answer that question, this study has the following sub-objectives: to survey the organizational structure of public hospital pharmacies in Norway and Pakistan; to compare the public hospital pharmacy practices in both countries; to determine the improvements in public hospital pharmacy practice in both countries; and to understand the management challenges faced by the public hospital pharmacists in both countries.

## 2. Materials and Methods

### 2.1. Study Design and Study Location

A pilot cross-sectional study was conducted to examine public hospital pharmacies’ structure and practices in two disparate countries. Norway and Pakistan were selected for the study. In the Institute for Health Metrics and Evaluation’s ranking of 195 nations’ healthcare systems, Norway holds second place [23]. By contrast, Pakistan ranks 154th among 195 countries in terms of quality and accessibility of healthcare. Pakistan is struggling to achieve healthcare on par with other developing countries by setting ambitious goals and expectations [24], but for now, these profound differences in healthcare quality provide a springboard for this study which can be used in Pakistan’s efforts to improve its pharmaceutical system. The Norwegian hospitals included in this study are Ullevål hospital (NH1) and Akershus University Hospital (NH2), while NICVD (PH1) and Sindh Government Hospital in Korangi (PH2) are the counterparts from Pakistan. These public hospitals were not randomly selected. Rather, they were chosen because the researchers have close links to them.

All the sampled hospitals are tertiary care hospitals with more than 400 beds. Their public mission, research, and teaching objectives are also similar. NH1 is the biggest state hospital for providing quality care and conducting research. The pharmacy leadership of NH1 is responsible for other public hospitals in Norway. In Pakistan, PH1 plays a pivotal role in caring for patients with heart disease. It is the first tertiary cardiac care institute in South Asia. At the time of this study, PH1 had 5 pharmacies and 15 pharmacists, while PH2 had 1 pharmacy and 2 pharmacists. NH1 had 1 pharmacy and 15 pharmacists, while NH2 had 1 pharmacy and 10 pharmacists.

### 2.2. Data Collection Tool

The structured questionnaire for the research was adapted from guidelines crafted by the ASHP regarding minimum standards for pharmacies in hospitals [25]. The ASHP guidelines on least standards provide the fundamentals for pharmacy services in U.S. hospitals. These recommendations describe a minimum level of facilities and services that a hospital pharmacy must deliver. Some of these recommendations might be appropriate to other healthcare settings or help in assessing the extent and quality of pharmacy services. Several researchers have used these standards to evaluate hospital pharmacy practices in Iran, Saudi Arabia, Lebanon, and Spain, respectively [26,27,28,29]. The questionnaire used in this study was modified from the original ASPH standards. The nine core elements of care recommended by ASHP were included. The ASPH guidelines used open-ended questions in subsections (formulary management, samples archiving system, inspection criteria, barcoding method, and method of sterilization), which we modified to closed-ended questions. Our structured questionnaire eventually comprised 11 sections with 63 “Yes/No” questions related to the following: (1) practice management; (2) pharmacy service availability; (3) workspace; (4) medication-use policy development; (5) optimizing medication therapy; (6) drug product procurement and inventory management; (7) preparing, packaging, and labeling medications; (8) medication dispensing and delivery; (9) monitoring medication use; (10) evaluating the effectiveness of the medication-use system; and (11) research.

After assembling the questionnaire, we arranged for two pharmacists from each country to review it for clarity. Modifications that were deemed necessary were incorporated into the final version of the questionnaire. This final version and a consent form were distributed to all pharmacists (17 Pakistani, 25 Norwegian) in the sampled hospitals. The study was completed in 2020. The total number of respondents was 12 (response rate = 28.6%).

### 2.3. Statistical Analysis

Data entry was performed in Microsoft Excel. Frequency tables were generated, and cross-tabulations were performed. Chi-square test was applied to analyze the differences between outcomes from hospital pharmacies in Pakistan and Norway at the level of significance (*p* < 0.05) using Statistical Package for the Social Sciences (SPSS version 23).

### 2.4. Ethical Considerations

There were no ethical considerations related to this study as it involved no internal data or information from the organization. After a brief introduction to the study, participants voiced their consent and filled out written informed consent forms.

## 3. Results and Discussion

Females (n = 4 (three Norwegian pharmacists and one Pakistani pharmacist)) constituted 33.3% of the sample, with the remaining 66.7% being eight males (three Norwegian pharmacists and five Pakistani pharmacists). The mean age of the participants was 37.08 (min: 25, max: 56) years. Over half of the sample (n = 8, 66.7%) were 35 years or younger, whereas 33.3% (n = 4) were 36 years or older. The mean number of practice years was 9.68 (min: 1.5 years; max: 29.2 years). More than half of the sampled physicians (n = 8, 66.7%) (four Pakistani and four Norwegian pharmacists) had 10 or fewer years of practice, while 33.3% (n = 4, (two Pakistani and two Norwegian pharmacists)) had 11 or more years of practice.

The main purpose of this research was to compare the organizational structure of public hospital pharmacies and their practices in Norway and Pakistan. This study noted the improvements in hospital pharmacies of both countries at government level. The challenges faced by hospital pharmacists and their managers in both countries were also addressed.

Hospital pharmacy is built on practice management standards, medication-use policy, optimizing medication therapy, drug procurement and inventory management, preparing, packaging, and labeling medications, medication dispensing and delivery, monitoring medication use, evaluating the effectiveness of the medication-use system, and research to improve services. These nine features, along with the two additional categories of pharmacy availability and workplace, formed the basis of the survey. The results are presented in the next 11 sections.

### 3.1. Practice Management

Table 1 below, as well as the subsequent 10 tables in the next sections, compares the success of Pakistan and Norway in implementing their pharmacy practice. Because two hospitals were surveyed in each country, and the pharmacists in each study hospital gave unanimous answers to their questionnaires, the only possible fraction of positive responses for each country is 0, 50, or 100 percent.

The mission of pharmacists is to help people make the best use of medications [30]. This study revealed that a written mission statement with goals and scope of services was found in both of the study hospitals in Pakistan as well as Norway. Practice standards and guidelines in the pharmacy were not found in Pakistan but were in Norway. Practice guidelines for several disease conditions and healthcare subjects exist in national organizations and specialist boards [31]. Improving as well as upholding a policy and procedure manual can deliver a well-organized and efficient approach to administering transformation [32]. Hiring a workforce amid reviewing or evolving policies and procedures can build self-esteem and contentment [33]. This study found that policy and procedure manuals were common in all the examined hospitals in Norway, while half those hospitals in Pakistan were found to be deficient. There must be a recognized, organized process for familiarizing new employees with the pharmacy department [34]. Clinical practice guidelines have been shown to improve processes of care, clinical outcomes, and quality medical care [35]. ASHP surveys conducted in 2007 and 2010 showed that 80% of U.S. hospitals used such policies and procedures regularly to minimize medication errors [14,36]. Yet, this study found no established procedure for orienting new pharmacists in Pakistan, unlike Norway which had well-established procedures in place. However, a designed job description of each position in the pharmacy department was found in half of the studied hospitals in Pakistan as well as Norway. Written work schedules were being used in both of the subject hospitals in Pakistan and only half those in Norway. Scheduling staff members in service industries can be more complicated than in manufacturing. In service industries, benefits and wages often comprise a significant fraction of outlays. From a competitive standpoint, staff planning becomes significant when fairness and staff preferences are crucial, customer needs are changing, and wages are the key factor [37].

### 3.2. Pharmacy Service Availability

Twenty-four-hour pharmacy services were not present in any of the studied hospitals in Pakistan but did exist in half the Norwegian hospitals. On the other hand, after-hours pharmacy access was available in half of the studied hospitals in both Pakistan and Norway (see Table 2). Round-the-clock facilities are essential in institutions that have clinical programs which require careful medication therapy. If it is not possible to provide 24 h service, then a pharmacist should be on an on-call basis [38].

Moreover, a significant difference (*p* < 0.05) was observed in various factors related to practice management between Pakistan and Norway, including personal safety education, pharmacist involvement in immunization, qualified pharmacy technicians, comprehensive pharmacy computer system integrated with computerized order entry, medication administration, and pharmacy system integrated with clinical decision support tools. Neuhauser et al. [39] compared the demographics, professional activities, and job satisfaction of immunization-certified and other pharmacists in Texas. This cross-sectional study found that significantly more certified pharmacists were involved in immunizations as an advocate, partner (hosting immunization providers in the practice), and provider (99%) when compared with non-certified pharmacists (24%; *p* < 0.001). Of those certified, 74% classified themselves as providers, actually administering immunizations. The most frequently administered vaccines were influenza (96%), pneumococcal (77%), hepatitis B (55%), and tetanus–diphtheria (19%).

### 3.3. Pharmacy Workspace

There is a minimum recommendation for the space needed for a hospital pharmacy based on the number of clinical departments and hospital beds (see Table 3). In larger hospitals, the recommended guideline for drug storage is 0.3 m^2^–0.4 m^2^ for each bed [40,41,42]. The layout and its outcome on practice reveal an important role in aspects of routine procedures. Organizations should be planned in a way to control future costs on upgrades to existing facilities. Successful institutional planning methods have been shown to decrease functioning inadequacies and may reduce expenses by up to 30% [43]. The pharmacy department of any hospital should plan for appropriate resources to permit the proper receiving, storage, and formulation of medicines so as to guarantee drug integrity as well as employees’ safety. Sufficient office and meeting areas should be accessible for administrative, educational, and training activities. Previously, many shortcomings were reported in spaces, equipment, services, and drug counseling, which the pharmaceutical care departments are supposed to offer in the Iranian hospitals [41]. The present study revealed that adequate pharmacy spaces were not present in any of the studied hospitals in Pakistan, while Norway had sufficient space. Significant differences (*p* < 0.05) were found in space for medication storage and preparation in all of the studied hospitals in Norway compared to Pakistan, where only 50% of the hospitals in Pakistan had adequate space. Adequate office and meeting areas for the pharmacy department were present in all studied hospitals of Pakistan as well as Norway. Adequate space and resources for drug information services were common in Norway but only half so in Pakistan.

### 3.4. Medication-Use Policy

Formulary controlling is key to regulating the quality and cost of pharmaceuticals. Therefore, any drug formulary system must be established on suitable clinical as well as pharmacoeconomic foundations [42,43]. A pharmacy and therapeutic committee operates as a consultative body to the medical workforce and managers in all subjects concerning medicines and supervises the formulary system. This practice has been defined as the process by which a healthcare institution develops strategies about the use of medications, pharmacotherapies, and drug-related products and detects those that are most medically suitable and economical to assist the health and welfare of a patient population [9]. The medication-use policy development section of the study revealed that there was no concept of a supervised formulary of accredited medications in the studied hospitals in Pakistan, while there is in Norway (see Table 4). The P&T committee regularly reviewed the formulary for information in 50% of studied hospitals of Norway while this concept was absent in the studied hospitals of Pakistan. Furthermore, there was a significant difference (*p* < 0.05) in that the P&T committee existed and operated according to guidelines in all studied hospitals of Norway.

### 3.5. Optimizing Medication Therapy

The section on optimizing medication therapy found significant differences (*p* < 0.05) between Pakistan and Norway with respect to providing direct patient care by the pharmacist, maintaining patient confidentiality, pharmacist’s ready access to overarching medication histories for all patients, and pharmacist involved in medication therapy decisions. The studied hospitals in Norway were found to be superior to Pakistan in the optimization of drug therapy (see Table 5). In the USA, it was reported that drug therapy for ambulatory patients taking multiple medications to treat chronic conditions could be improved through collaboration between physicians and community pharmacists [44].

### 3.6. Drug Product Procurement and Inventory Management

With increased understanding of the proper delivery of medicine, keeping a record of drug distribution is recommended by the Food and Drug Administration [45]. The standards concerning drug procurement and inventory management were found to be outstanding in the studied hospitals of Norway (see Table 6). Because pharmaceutical purchasing is a multidisciplinary procedure requiring medical, pharmaceutical, administrative, financial, and, often, political skills, it should be carried out according to the laws and procedures recommended by international bodies. Migbaru et al. [46] and Nigah et al. [47] also reported the need for proper management of inventory control in hospital pharmacies in Ethiopia and India, respectively.

### 3.7. Preparing, Packaging and Labeling Medications

Good dispensing practices ensure that medication is delivered to patients with appropriate directions in a pack which preserves the potency of the drug up to the time of use [48]. Compounding, then, is akin to the preparation of unlicensed drugs, both sterile and non-sterile, with the intention to fulfill patient-specific needs which are not covered by licensed medications. Compounded drugs are usually prepared extemporaneously in the community as well as in hospital pharmacies for custom orders [49]. According to Fadel et al. [50], minimum standards and best practice recommendations to ensure the safety of sterile compounding were reported in hospitals in Saudi Arabia, the United Arab Emirates, Bahrain, Kuwait, Egypt, Malta, the USA, and others. However, advanced technologies were not implemented by the majority of the hospitals. This study found that the Pakistani hospitals had no compounding facilities, sterile preparation areas, unit dose dispensation for admitted patients, and unit doses packaged with a barcode (see Table 7).

### 3.8. Medication Dispensing and Delivery

Pharmacists must have instant access to the patient’s diagnosis as well as the expected pharmacotherapeutic or medical purposes of drugs. Half the studied hospitals in both Pakistan and Norway revealed that pharmacists had such access. Spoken drug orders must be given only in extraordinary and emergency situations. In such cases the order should be repeated back to the prescriber to confirm it, and written confirmation must be presented to the pharmacy within a specified period [51]. Documented policy and procedure for spoken medication orders existed in only half the studied hospitals of Pakistan, while Norway was fully compliant—at least in the two studied hospitals. All medication orders should be prospectively reviewed by a pharmacist and evaluated concerning their relevance to patient and clinical outcomes before dispensation of the first dose, unless there is an emergency [51]. However, the study found that pharmacists reviewed medication orders in 50% of the subject hospitals in Pakistan, and in all the Norwegian counterparts. There were well-defined policies and procedures for medication delivery and administration in all of Norway’s studied hospitals, and no such practice was found in either of the two hospitals in Pakistan (see Table 8). In France, it was reported that a wide range of errors occurred during the dispensing process following ASHP practice guidelines [52].

### 3.9. Monitoring Medication Use

Fifty per cent of the studied hospitals in Pakistan had pharmacists conducting medication therapy monitoring, while this practice was common in all the studied hospitals of Norway. However, in 50% of the studied hospitals of Pakistan, pharmacists (*p* < 0.05) were involved in educating and counseling patients, while this practice was not found in Norway (see Table 9).

According to Saffouh El Hajj et al. [53], the lowest incidence of agreement across the drug procurement, distribution process statements, and monitoring medication use was observed for the adequacy of medication supplies statements (33% of all respondents). Medication supply statements are used to indicate whether the medication supplies within the country are adequate or if shortages are common in the country.

### 3.10. Evaluating the Effectiveness of the Medication-Use System

Drug shortages have been recognized as a public health problem in several regions around the world. They can impair the quality and effectiveness of patient care, and increase the cost of therapy [54]. Therefore, there must be well-defined policies and procedures to cope with shortages of medications. However, significant differences (*p* < 0.05) were found between Pakistan’s and Norway’s studied hospitals in the documentation of pharmacist-provided care services, ongoing programs for supervising drug consumption and costs, policies and procedures for optimal use of antimicrobial agents, pharmacists monitoring patients’ laboratory reports of microbial considerations or appropriate diagnostic markers, and suggesting prescribers (see Table 10). Pharmacists along with other hospital personnel should find and frequently modify strategies concerning medication error and adverse drug event interception and documenting [54,55,56]. However, this study revealed that there was no process to routinely observe and document the workload and performance of the pharmacy department in any of the studied hospitals from Pakistan, and only half of those in Norway. There was an existing program for observing drug consumption and costs in 50% of Pakistan’s studied hospitals, and the same was true in Norway (100%). Pharmacists were involved in multidisciplinary efforts to avert, sense, and settle drug-related problems in 50% of the studied hospitals in Pakistan, whereas in Norway, all the pharmacists in all the studied hospitals carried out this activity. Similarly, pharmacists were involved in strategies and methodologies concerning medication error and adverse events in 50% of the studied hospitals in Pakistan, and all the studied hospitals in Norway. Hospital patients might need constant observation of their drug therapy and effects to deal with new or recurring drug-associated problems. Whether for a new problem or subsequent monitoring, the pharmacist evaluates the particular pharmacotherapeutic problem in light of the overall medical and drug history [54]. In Oslo, a hospital pharmacy operating group was established to deal with shortages of medications in all of the hospital regions. As a result, these shortages were controlled efficiently [56]. In Italy, the introduction of policies and procedures for optimal use of antimicrobial agents resulted in significant decreases in adverse events [57]. According to Chukwuani et al. [58], noticeable gaps in knowledge regarding rational drug use still exist among the cadres of healthcare professionals in Nigeria.

### 3.11. Research

As with any discipline, research is vital for the development of hospital as well as clinical pharmacy service. The pharmacist must instigate, contribute to, and encourage clinical as well as practice-associated research that supports the aims and intentions of an institution [59]. The study found that pharmacists participating in and supporting clinical and practice-related research relevant to the goals, purposes, and assets of the particular hospital were significantly different (*p* < 0.05) in the studied hospitals of Pakistan, unlike Norway where all the questioned pharmacists were involved in such activities (see Table 11). According to Moles [60], clinical pharmacy in Australia has been guided through the development and update of the Standards of Practice for Clinical Pharmacy, published in 2013, and pharmacists actively participate in research-oriented programs and activities. However, poor implementation of research-oriented policies in hospital pharmacies was found in Middle East countries [61].

## 4. Limitations of the Study

This study has several limitations. The first limitation was that the study used a convenience sampling method. Secondly, the results of the study were limited to the sampled hospitals. Thirdly, the limitations of pilot cross-sectional studies and convenience sampling can apply in this case [62]. Fourthly, a low response rate was received from the participating pharmacists.

## 5. Conclusions and Recommendations

This pilot study compared healthcare services and practices in hospital pharmacies in Pakistan and Norway. The evaluation was based on the ASHP guidelines for the systematic assessment of minimum standards in hospital pharmacies. These guidelines are built upon several standards regarding practice management, medication-use policy development, optimizing medication therapy, drug product procurement and inventory management, preparing, packaging, and labeling medications, dispensing and delivery of medications, monitoring medication use, evaluating the effectiveness of the medication-use system, and research to improve the provision of services.

After surveying for all of these standards, it was found that the studied hospital pharmacies in Norway had better utilization of practice management standards than those in Pakistan. This study was the first of its kind to explore differences and similarities between hospitals in Pakistan and Norway. It found that the quality and degree of services provided by hospital pharmacies in Pakistan was not up to the mark. Therefore, there is a need for states, NGOs, academic institutions, hospitals, and other health-related organizations to understand the importance of hospital pharmacy practices in Pakistan, so as to deliver quality services and ensure patient safety. Furthermore, this study identified opportunities for improving and expanding the services provided by the pharmacy departments in both Pakistan and Norway. An international collaboration between countries could advance the provision of pharmacy services and ensure that patients receive the care they deserve.

Based on the study outcomes, our proposed solutions are labor- and resource-intensive and would require standardization and oversight. Accurate medication lists across the healthcare continuum are of paramount importance. It is also suggested that pharmacy practice could be improved through cooperation, sharing successes, providing examples of specialized pharmacy services, and encouraging student exchanges. Moreover, the Ministry of National Health Services in Pakistan could improve hospital pharmacy services through the following steps: first, unfreeze the existing situation, which can be achieved by showing that the reasons for change outweigh any arguments against change; second, transition the system to a new equilibrium; and finally, freeze the new system, thereby ensuring that people do not revert to the old ways.

## Figures and Tables

**Table 1:** Pharmacists’ assessments on practice management.

	Pakistan (%)	Norway (%)
Written mission statement, goals and scope of services present	100	100
Practice standards and guidelines in the pharmacy	0	100
Pharmacy policy and procedure manual	50	100
Designed job description of each position in the pharmacy	50	50
Established, structured procedure for orienting new personnel	0	100
Written work schedules	100	50
Procedure for measuring performance of pharmacy employees	50	100
Defined policy regarding conflict-of-interest and ethical conduct	0	100

**Table 2:** Pharmacists’ assessments on pharmacy service availability.

	Pakistan (%)	Norway (%)
24 h pharmacy services	0	50
After-hours pharmacy access in the absence of 24 h	50	50
Personal safety education	100	100
Pharmacy department participation in emergencies	100	100
Pharmacist involved in immunization	0	0
Director of pharmacy services	50	50
Qualified pharmacist present	100	100
Qualified pharmacy technicians present	0	100
Non-qualified pharmacy technicians present	100	100
Comprehensive pharmacy computer system integrated with computerized provider-order-entry medication administration, electronic health record, and patient billing systems	0	100
Pharmacy system is integrated with clinical decision support tools	0	50
Pharmacist actively participate in hospital and health-system committees	100	100

**Table 3:** Pharmacists’ assessments on pharmacy space and other resources.

	Pakistan (%)	Norway (%)
Adequate pharmacy spaces and resources as per any standard	0	100
Adequate space for medication storage and preparation as per standard	50	100
Patient assessment and consultation area	50	100
Adequate office and meeting areas	100	100
Adequate space and resources for drug information services	50	100

**Table 4:** Pharmacists’ assessments on medication-use policy development.

	Pakistan (%)	Norway (%)
Well-controlled formulary of approved medications	0	100
Pharmacist provides patient specific information about drugs and drug therapy to health professionals, patients, and patients’ caregivers	100	100
Pharmacist ensures timely dissemination of drug information	100	100
P&T committee regularly reviews the formulary for safety information	0	50
P&T committee exists and operates according to guidelines	50	100

**Table 5:** Pharmacists’ assessments on optimizing medication therapy.

	Pakistan (%)	Norway (%)
Pharmacists provide direct patient care	50	100
Patient’s confidentiality is maintained	50	100
Pharmacists have immediate access to comprehensive medication histories for each patient	50	50
Pharmacists provide oral and written consultations	50	50
Recommendations made by the pharmacist and actions taken in response to those recommendations are documented	50	100
Pharmacists involved in medication therapy decisions	100	100

**Table 6:** Pharmacists’ assessments on drug product procurement and inventory management.

	Pakistan (%)	Norway (%)
Policies and procedures for managing medication acquisition	50	100
Criteria for selecting drug product manufacturers and suppliers	50	100
Proper medication storage conditions	50	100
Policies and procedures for managing drug product shortages	0	100
Policies and procedures for distribution and use of controlled substances	50	100
Policies and procedures for patient’s own medications	0	50
Policies and procedures for inspection of all stocks of medications	50	100
Policies and procedures for returning recalled, expired, and other unusable items	100	100

**Table 7:** Pharmacists’ assessments on preparing, packaging, and labeling medications.

	Pakistan (%)	Norway (%)
Compounding facility available	0	100
Area for sterile preparations available	0	100
Unit dose dispensation for admitted patients	0	50
Unit dose packaged with a barcode	0	100

**Table 8:** Pharmacists’ assessments on medication dispensing and delivery.

	Pakistan (%)	Norway (%)
Pharmacist has immediate access to the patient’s diagnosis	50	50
Documented policy and procedure for spoken medication orders	50	100
Medication orders are reviewed by pharmacist	50	100
Policies and procedures for medication delivery and administration	0	100

**Table 9:** Pharmacists’ assessments on monitoring medication use.

	Pakistan (%)	Norway (%)
Pharmacist conducts medication therapy monitoring	50	100
Pharmacist is involved in educating and counseling patients	50	0

**Table 10:** Pharmacists’ assessments on evaluating the effectiveness of the medication-use system.

	Pakistan (%)	Norway (%)
Documentation of pharmacist provided patient care services	50	50
Process to routinely monitor and document workload and financial performance	0	50
Ongoing program for monitoring drug utilization and costs	50	100
Pharmacist involved in multidisciplinary efforts to prevent, detect, and resolve drug-related problems	50	100
Pharmacist involved in policies and procedures regarding medication error and adverse event	50	100
Policies and procedures for optimal use of antimicrobial agents	50	100
Pharmacist monitors patients’ laboratory reports of microbial-sensitivities or applicable diagnostic markers and advise prescribers	50	100
Pharmacist participates in antimicrobial stewardship and infection-prevention	50	100

**Table 11:** Pharmacists’ assessments on research.

	Pakistan (%)	Norway (%)
Hospital supports clinical and practice-related research appropriate to its goals, objectives, and resources	50	100

## Data Availability

The data presented in this study are available on request from the corresponding author. The data are not publicly available.
